# Polymer degrading marine *Microbulbifer* bacteria: an un(der)utilized source of chemical and biocatalytic novelty

**DOI:** 10.3762/bjoc.20.146

**Published:** 2024-07-17

**Authors:** Weimao Zhong, Vinayak Agarwal

**Affiliations:** 1 School of Chemistry and Biochemistry, Georgia Institute of Technology, Atlanta, GA 30332, USA; 2 School of Biological Sciences, Georgia Institute of Technology, Atlanta, GA 30332, USA

**Keywords:** bacteria, hydrolases, marine natural products

## Abstract

*Microbulbifer* is a genus of halophilic bacteria that are commonly detected in the commensal marine microbiomes. These bacteria have been recognized for their ability to degrade polysaccharides and other polymeric materials. Increasingly, *Microbulbifer* genomes indicate these bacteria to be an untapped reservoir for novel natural product discovery and biosynthetic novelty. In this review, we summarize the distribution of *Microbulbifer* bacteria, activities of the various polymer degrading enzymes that these bacteria produce, and an up-to-date summary of the natural products that have been isolated from *Microbulbifer* strains. We argue that these bacteria have been hiding in plain sight, and contemporary efforts into their genome and metabolome mining are going to lead to a proliferation of *Microbulbifer*-derived natural products in the future. We also describe, where possible, the ecological interactions of these bacteria in marine microbiomes.

## Introduction

Microbial natural products represent an important reservoir for drug discovery and along with their (semi)synthetic derivatives, constitute a major component of the contemporary pharmaceutical arsenal. Their thusly validated utility is juxtaposed against the barriers to natural product discovery; high rediscovery rates, reliance on largely serendipitous response in bioactivity assays, and the resources and expertise required for their structure determination being the primary impediments [1–2]. With this background, accessing biological sources that have not yet been extensively mined for natural product discovery is a promising route, one which, at the very least, promises to ameliorate the problem of continued rediscovery of known natural products. This review outlines the recent progress that has been realized using bacteria of the genus *Microbulbifer*. Together with the emerging field of natural product discovery from *Microbulbifer* bacteria, this genus of obligate marine bacteria is well validated to be a valuable source of biopolymer degrading enzymes. In this review, we outline the discovery of *Microbulbifer* enzymes that have substantial biocatalytic potential as it pertains to biopolymer degradation, followed by a description of natural product discovery from this genus.

**Figure 1:**
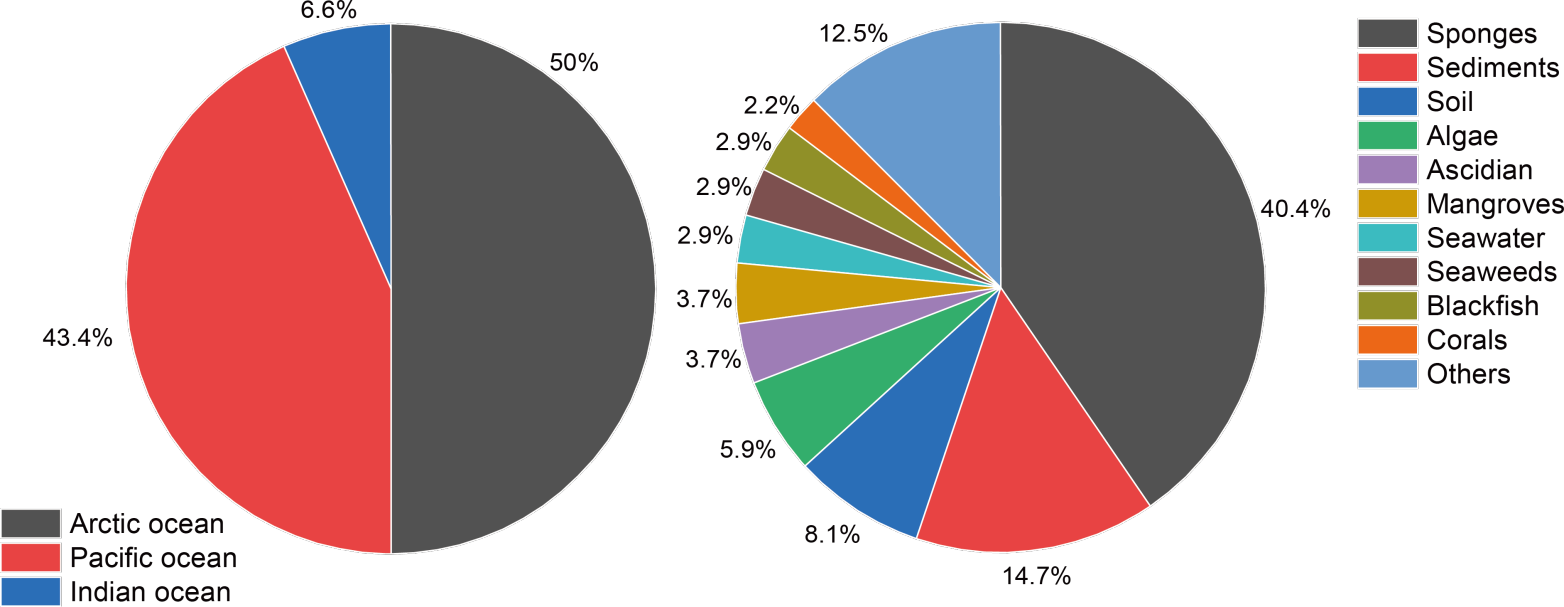
Oceanic distribution and marine holobiont sources of *Microbulbifer* strains described in the literature.

*Microbulbifer* is a genus of rod-shaped, aerobic Gram-negative *Alteromonadaceae* family γ-proteobacteria. They are halophilic obligate marine bacteria that are frequently isolated from diverse marine holobionts such as sponges [3–4], sediments [5], algae [6], and corals [7] using media that mimic the native salt water habitat. Strains of this genus are detected together with *Pseudovibrio* and *Ruegeria* strains that grow in identical culture conditions, and hence obtaining axenic *Microbulbifer* strains may require repeated rounds of serial enrichment. This genus was first described by Gonzales et al. in 1997 [5]. More than 130 strains have been reported from regions of wide geographical dispersion (Table 1, Figure 1). *Microbulbifer* bacteria are well known for their capacity to degrade not only a wide variety of polysaccharides including cellulose, agar, chitin, alginate, and xylan [8–11], but also plastics [4,12]. Progressing from their isolation and cultivation, natural product discovery from *Microbulbifer* bacteria is starting to gather steam [6,13–14]. The major classes of natural products isolated from *Microbulbifer* to date include alkaloids [15], fatty acid and polyketides [16–17], and non-ribosomally synthesized peptides [3–4,7]. These findings validate the potential for secondary metabolite discovery from *Microbulbifer* genus. This review will cover the distributions and origins of *Microbulbifer* bacteria strains, degradation enzymes, and secondary metabolite discoveries. A focus is placed on the novel chemical structures reported with reference to their biological activities and the biosynthetic studies they have inspired.

**Table 1:** Summary of marine sources, geographical locations, and key characteristics of *Microbulbifer* strains reported in literature.

		Origin	Location	Key characteristics	References

1	*Microbulbifer hydrolyticus* gen. nov.	marine pulp mill	USA	produces hydrolytic enzymes, polyethylene degradation	[5,18–19]
2	*Microbulbifer* degradans. 2-40	salt marsh	USA	degrades polysaccharides, polyserine linkers, chitinase	[20–22]
3	*Microbulbifer salipaludis* sp. nov.	salt marsh	Korea	–^a^	[8]
4	*Microbulbifer arenaceous* sp. nov.	red sandstone	Scotland	hydrolyzes chitin, esculin, gelatin, and starch	[23]
5	*Microbulbifer elongates* comb. nov.	–^a^	Germany	–^a^	[24]
6	*Microbulbifer* sp. JAMB-A7	sediment	Japan	β-agarase	[25]
7	*Microbulbifer* sp. JAMB-A94	sediment	Japan	β-agarase, ι-carrageenase	[10,26–29]
8	*Microbulbifer* sp. JAMB-A3	sediment	Japan	β-agarase	[10,30]
9	*Microbulbifer maritimus* sp. nov.	sediment	Korea	–^a^	[31]
10	*Microbulbifer* sp. A4B-17	ascidian	Palau	produces 4-HBA and parabens	[6]
11	*Microbulbifer* sp. A4A-72	sponge	Palau	produces 4-HBA	[6]
12	*Microbulbifer* sp. V296	sponge	Yap	produces 4-HBA	[6]
13	*Microbulbifer* sp. A4B-20	algae	Palau	produces 4-HBA	[6]
14	*Microbulbifer* sp. A2C-113	sponge	Palau	produces 4-HBA	[6]
15	*Microbulbifer* sp. A4A-79	ascidian	Palau	produces 4-HBA	[6]
16	*Microbulbifer* sp. AM222	sediment	Palau	produces 4-HBA	[6]
17	*Microbulbifer* sp. AM292	sediment	Palau	produces 4-HBA	[6]
18	*Microbulbifer* sp. A4A-68	sponge	Palau	produces 4-HBA	[6]
19	*Microbulbifer* sp. A2D-17	sponge	Palau	produces 4-HBA	[6]
20	*Microbulbifer* sp. I1876	algae	Japan	produces 4-HBA	[6]
21	*Microbulbifer* sp. C91	algae	Yap	produces 4-HBA	[6]
22	*Microbulbifer* sp. HG111	sponge	Japan	produces 4-HBA	[6]
23	*Microbulbifer* sp. F-104	algae	Palau	produces 4-HBA	[6]
24	*Microbulbifer* sp. V32	sponge	Yap	produces 4-HBA	[6]
25	*Microbulbifer* sp. HG125	sponge	Japan	produces 4-HBA	[6]
26	*Microbulbifer* sp. A3G-2	ascidian	Palau	produces 4-HBA	[6]
27	*Microbulbifer* sp. A3P2-23-3-2	ascidian	Palau	produces 4-HBA	[6]
28	*Microbulbifer* sp. AM220	sediment	Japan	produces 4-HBA	[6]
29	*Microbulbifer* sp. YM3-0188	sponge	Palau	produces 4-HBA	[6]
30	*Microbulbifer* sp. A2D-15	sediment	Palau	produces 4-HBA	[6]
31	*Microbulbifer* sp. HG869	ascidian	Palau	produces 4-HBA	[6]
32	*Microbulbifer* sp. HG279	sponge	Japan	produces 4-HBA	[6]
33	*Microbulbifer* sp. ssthio04PA2-36a	sponge	Palau	produces 4-HBA	[6]
34	*Microbulbifer celer* sp. nov.	saltern	Korea	–^a^	[32–33]
35	*Microbulbifer halophilus* sp. nov.	saline soil	China	–^a^	[34]
36	*Microbulbifer* sp. CMC-5	seaweed	India	degrades polysaccharides, β-agarase	[35–37]
37	*Microbulbifer variabilis* sp. nov.	algae	Japan	–^a^	[38]
38	*Microbulbifer epialgicus* sp. nov.	algae	Japan	–^a^	[38]
39	*Microbulbifer* sp. L4-n2	sponge	France	produces parabens	[13]
40	*Microbulbifer donghaiensis* sp. nov.	sediment	China	–^a^	[39]
41	*Microbulbifer chitinilyticus* sp. nov.	mangrove	Japan	chitin-degrading	[9]
42	*Microbulbifer okinawensis* sp. nov.	mangrove	Japan	chitin-degrading	[9]
43	*Microbulbifer* sp. SD-1	seawater	Korea	degrades agar	[40]
44	*Microbulbifer* sp. JAM-3301	sediment	Japan	inulinase producer	[41]
45	*Microbulbifer maritimus*	seaweed	India	agarase producer	[42]
46	*Microbulbifer* sp. 6532A	seaweed	Japan	alginate lyase producer	[43–44]
47	*Microbulbifer marinus* sp. nov.	sediment	China	–^a^	[45]
48	*Microbulbifer yueqingensis* sp. nov.	sediment	China	–^a^	[45]
49	*Microbulbifer gwangyangensis* sp. nov.	tidal flat	Korea	–^a^	[46]
50	*Microbulbifer pacificus* sp. nov.	sponge	Korea	–^a^	[46]
51	*Microbulbifer mangrove* sp. nov.	mangrove	India	polysaccharide-degrading	[11]
52	*Microbulbifer elongatus* HZ11	seawater	China	seaweed-degrading	[47]
53	*Microbulbifer thermotolerans* DAU221	sediment	Korea	produces carbohydrate esterase, α-amylase, esterase, chitinase, GH3 -glucosidase polysaccharide lyase	[48–53]
54	*Microbulbifer elongatus* sp. A13	seaweeds	India	agarase producer	[54]
55	*Microbulbifer rhizosphaerae* sp. nov.	halophytic plant	Spain	–^a^	[55–56]
56	*Microbulbifer* sp. ALW1	algae	China	produces alginate lyase, laminarinase, β-glucosidase, chondroitinase	[12,57–62]
57	*Microbulbifer flavimaris* sp. WRN-8	sediment	China	–^a^	[63]
58	*Microbulbifer mangrovistrain* DD-13	mangrove	India	degrades polysaccharides	[64]
59	*Microbulbifer echini* sp. AM134	sea urchin	Korea	–^a^	[65]
60	*Microbulbifer aggregans* sp. CCB-MM1	sediment	Malaysia	produces sulfite reductase	[66–68]
61	*Microbulbifer aestuariivivens* sp. GHTF-23	tidal flat	Korea	–^a^	[69]
62	*Microbulbifer* sp. Q7	sea cucumber	China	produces β-agarase, alginate lyases	[70–72]
63	*Microbulbifer* sp. 127CP7-12	sponge	Korea	produces violacein	[73]
64	*Microbulbifer hydrolyticus* IRE-31-192	chemical mutation	China	polyethylene degradation	[74]
65	*Microbulbifer* sp. GL-2	blackfish	Japan	cellulase	[75]
66	*Microbulbifer agarilyticus*. GP101	invertebrate turbo cornutus	Korea	–^a^	[76]
67	*Microbulbifer* sp. BN3	sediment	China	β-agarase, chitinase	[77–79]
68	*Microbulbifer* sp. WMMC-695	tunicate	USA	produces antibacterial acetamido-4-hydroxybenzoate esters	[14]
69	*Microbulbifer* sp. BH-1	seawater soil	China	β-agarase	[80]
70	*Microbulbifer* sp. C4-6	coral	Japan	produces fatty acid with weak growth inhibition against *Saccharomyces cerevisiae*	[16]
71	*Microbulbifer* sp. AG1	mangrove soil	China	β-agarase producer	[81]
72	*Microbulbifer* sp. KIT−19	seawater	Japan	alginate lyase producer	[82]
73	*Microbulbifer harenosus* sp. nov.	coastal sand	China	alginate-degrading	[83]
74	*Microbulbifer* sp. DC3-6	coral	Japan	produces antimicrobial and cytotoxic alkanoyl imidazoles	[15]
75	*Microbulbifer* sp. SH-1	coastal soil	China	alginate lyase producer	[84]
76	*Microbulbifer elongates*. PORT2	seawater	Indonesia	agarase producer	[85–86]
77	*Microbulbifer hainanensis* sp. nov.	sediment	China	–^a^	[87]
78	*Microbulbifer* sp. YNDZ01	macroalgae	Indonesia	lipase, ι-carrageenase	[88–89]
79	*Microbulbifer* sp. CL37	sediment	Malaysia	xylanase producer	[90]
80	*Microbulbifer* sp. SOL66	soil	Korea	degrades poly(3-hydroxybutyrate)	[91–92]
81	*Microbulbifer* sp. SOL03	soil	Korea	degrades poly(3-hydroxybutyrate)	[91,93]
82	*Microbulbifer* sp. SOL51	soil	Korea	degrades poly(3-hydroxybutyrate)	[91]
83	*Microbulbifer* sp. SOL55	soil	Korea	degrades poly(3-hydroxybutyrate)	[91]
84	*Microbulbifer* sp. SOL84	soil	Korea	degrades poly(3-hydroxybutyrate)	[91]
85	*Microbulbifer* sp. GL-1	blackfish	Japan	cellulase-producing	[94]
86	*Microbulbifer* sp. GL-2	blackfish	Japan	cellulase-producing	[94]
87	*Microbulbifer* sp. GL-3	blackfish	Japan	cellulase-producing	[94]
88	*Microbulbifer* sp. YPW1	sediments	China	–^a^	[95]
89	*Microbulbifer* sp. SH-1	soil	China	alginate lyase producer	[96]
90	*Microbulbifer okhotskensis* sp. КMM 9862	sediment	Russia	–^a^	[97]
91	*Microbulbifer* sp. BY17	gracilaria	China	alginate lyase producer	[98]
92	*Microbulbifer* sp. YX04	mangrove	China	algicidal	[99]
93–130	*Microbulbifer* sp.	sponge	USA	–^a^	[100]
131	*Microbulbifer* sp. C10-1	coral	Japan	produces cyclic hexapeptides	[7]
132	*Microbulbifer* sp. RZ01	coastal environment	China	algicidal	[17]
133	*Microbulbifer* sp. TB12003	coastal environment	China	nonalgicidal	[17]
134	*Microbulbifer* sp. MLAF003	sponge	USA	produces cyclic hexapeptides	[4]
135	*Microbulbifer* sp. VAAF005	sponge	USA	produces cyclic hexapeptides	[4]
136	*Microbulbifer* sp. MKSA007	sponge	USA	produces linear peptides	[3]

^a^No information available.

## Review

### Biopolymer degrading enzymes from *Microbulbifer*

#### Agarases

**Figure 2:**
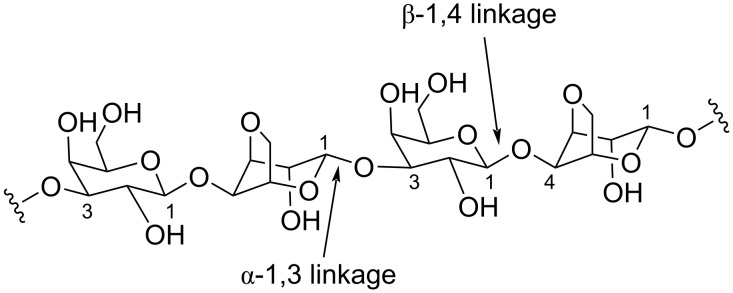
The chemical structure of agarose with the key β-1,4 linkage denoted.

Agarose is a natural polymer consisting of a linear chain of alternating residues of 3-*O*-linked β-ᴅ-galactopyranose and 4-*O*-linked 3,6-anhydro-α-ʟ-galactose. Agarose is a major component of agar, a polysaccharide present in the cell walls of some red algae [101]. Agarases are glycoside hydrolases (GHs) that degrade agarose to smaller oligosaccharides [102]. Marine bacteria of diverse genera produce agarases [103–105]. Based on the reactions that they catalyze, bacterial agarases are grouped as α- and β-agarases wherein they hydrolyze α-1,3 linkages and β-1,4 linkages in agarose, respectively (Figure 2). However, based on sequence similarity, agarases are alternatively classified into different families of glycoside hydrolases (GHs): GH-96 and GH-117 for α-agarases, and GH-16, GH-50, GH-86, and GH-118 for β-agarases [106]. All agarases characterized from *Microbulbifer* bacteria belong to β-agarases.

**GH-16 agarases:** The first report for the characterization of an agarase from the *Microbulbifer* genus was from the strain *Microbulbifer* sp. JAMB-A7 isolated from deep-sea sediment [25] wherein the agarase encoding gene *agaA7* was sequenced and cloned. The recombinant enzyme RagaA7 was produced in a *Bacillus subtilis* host and characterized to be a neoagarotetraose-producing GH-16 family endo-type β-agarase with a pH and temperature optima being 7.0 and 50 °C, respectively. Another thermostable neoagarotetraose-producing GH-16 endo-type β-agarase rAgaA was identified and cloned from deep-sea-derived *Microbulbifer* sp. JAMB-A94 with pH and temperature optima being 7.0 and 55 °C, respectively [26]. The recombinant enzyme was likewise produced using a *B. subtilis* host. The crystal structure of the catalytic domain was determined to show a β-jelly roll fold with structural similarity to two other β-agarases, ZgAgaA and ZgAgaB, from *Zobellia galactanivorans*, which is a model marine bacterium for the bioconversion of algal biomass [29,107–109]. The crystal structure suggested that the thermostability of rAgaA likely derives from the numerous surface salt bridges, relatively short length of the surface loops, and the increased number of Pro and Arg residues [29].

A truncated β-agarase gene without the carbohydrate-binding modules (Aga16A-ΔCBM) from the marine *Microbulbifer* sp. BH-1 was cloned and expressed in *Escherichia coli* with pH and temperature being 8.0 and 55 °C, respectively. The biocatalytic utility of the enzyme was also evaluated; it was used for neoagarooligosaccharide production in high yield [80].

Five additional GH-16 agarases AgaA3 produced in *B. subtilis*, ID2563 produced in *E. coli*, N3-1 produced in *Pichia pastoris*, AG1 produced in *E. coli*, and AgaF16A produced in *E. coli*, were cloned and expressed from marine *Microbulbifer* bacteria *Microbulbifer* sp. JAMB-A3 [30], *Microbulbifer* sp. Q7 [70], *Microbulbifer* sp. BN3 [77], *Microbulbifer* sp. AG1 [81], and *Microbulbifer elongatus* PORT2 [86], respectively. These agarases similarly degraded agarose into neoagarotetraose. With a view towards their biotechnological utility, AgaA3 was stable to high concentrations of surfactants.

**GH-50 agarases:** The strain *Microbulbifer elongatus* PORT2 was reported to be isolated in Indonesia [85]. In the sequenced genome of this bacterium were detected three agarases to be encoded which belonged to the GH-50 agarase family. The enzymes were named AgaA50, AgaB50, and AgaC50. Biochemical characterization of these three enzymes revealed that AgaA50 and AgaC50 generated neoagarobiose products implying cleavage within the agarose polymer (endolytic hydrolysis). The activity of AgaB50 was different, in that this enzyme cleaved at the external periphery of the agarose polymer to generate neagarosetetraose and neoagarobiose.

**GH-86 agarases:** The *Microbulbifer* sp. JAMB-A94 was shown to produce a GH-16 agarase (rAgaA) [26]. A gene encoding (*agaO*) for a novel GH-86 family agarase was also reported from the same bacterium [27]. The recombinant enzyme (rAgaO) was expressed in *B. subtilis* cells to demonstrate that this enzyme was an endo-type β-agarase and degraded agarose to neoagarohexaose as the main product [27]. The enzyme rAgaO represents the first and only report on the characterization of a purified agarase belonging to the family GH-86 from *Microbulbifer* bacteria.

#### Alginate lyases

**Figure 3:**
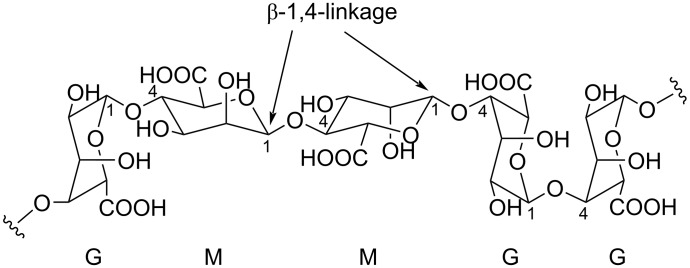
The chemical structure of the biopolymer alginate.

The polysaccharide alginate is produced by various seaweeds and bacteria [44]. Two building blocks, β-ᴅ-mannuronic acid (M) and α-ʟ-guluronic acid (G), are used to assemble alginate in homo- and heteropolymeric forms. The 1,4-*O*-linkage in alginate is cleaved by alginate lyases, in a β-elimination manner (Figure 3). Alginate lyases can be either endolyases, or exolyases with preference for polyM or polyG present in the alginate matrix [110]. Alginate lyases have been isolated from seaweed epiphytic microorganisms, mostly marine bacteria, including the *Microbulbifer* genus [111].

An alginate lyase encoding gene *AlgMsp* was discovered from the *Microbulbifer* sp. 6532A which was isolated from seaweed surfaces [44]. Recombinant AlgMsp expressed in *E. coli* was characterized to a slight preference for polyG over polyM substrates [44]. Recombinant alginate lyase AlyM expressed in *E. coli* from *Microbulbifer* sp. Q7 exhibited preference for polyG [71]. AlyM preferably degraded the glycosidic bond at the G–X linkage in alginate [71]. Further studies focused on the improvement in thermal stability by introduction of disulfide bonds [112–113].

Two new alginate lyases, AlgL17 and AlgL6, both expressed in *E. coli*, were characterized from *Microbulbifer* sp. ALW1 isolated from rotten brown algae [61–62]. AlgL17 preferentially degraded polyM with poor activity towards polyG, indicating it to be a polyM-specific alginate lyase. AlgL17 was an exotype alginate lyase based on its ability of producing 4-deoxy-ʟ-erythro-5-hexoseulose uronic acid (DEH) from sodium alginate [62]. AlgL6 degraded polyM, polyG, and sodium alginate in an exolytic manner. It exhibited good stability in the presence of some nonionic detergents [61].

Additional alginate lyases AlgSH17, BY17PV7, and MtAl138 were cloned and expressed in *E. coli* from *Microbulbifer* sp. SH-1 [96], *Microbulbifer* sp. BY17 [98], and *M. thermotolerans* DAU221 [52], respectively. Recombinant AlgSH17 exhibited both exolytic and endolytic activities [96]. BY17PV7 demonstrated wide substrate tolerance and good degradation effects for both polyM and polyG [98]. MtAl138 belongs to the polysaccharide lyase family 7 and is an endotype enzyme that produces di-, tri-, or tetrasaccharides from polyG and polyM [52].

#### Chitinases

**Figure 4:**
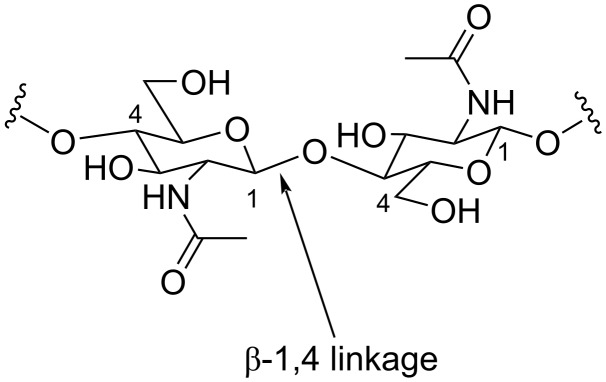
The chemical structure of chitin.

Chitin is a linear β-1,4-linked homopolymer of *N*-acetyl-β-ᴅ-glucosamine (GlcNAc), the second most abundant biomass on earth after cellulose (Figure 4). Approximately 10^11^ tons of chitin are produced annually in nature, about 70% of which originate from the ocean. Most of the chitin is recycled by bacteria and fungi as carbon and nitrogen sources [114–115]. Chitin is traditionally degraded with environmentally unfriendly concentrated acids or alkalis. Chitinases are hydrolytic enzymes that degrade glycosidic bonds between chitin polymers and belong to the GH-18 and GH-19 family [116]. Chitinases can be also classified as endo- or exochitinases.

Genomic analysis of a marine *Microbulbifer degradans* 2-40 revealed three chitin depolymerases (ChiA, ChiB, and ChiC) [117]. ChiB was cloned and expressed in *E. coli* [22]. It is a modular protein that is predicted to contain two GH-18 catalytic domains, two polyserine domains, and an acidic repeat domain. It functions as an exochitinase. The two catalytic domains have different activities on chitooligosaccharides. Each domain was maximally active from 30 °C to 37 °C and from pH 7.2 to 8.0. Both domains function cooperatively to degrade chitin [22]. It should be noted that *M. degradans* 2-40 was reclassified as *Saccharophagus degradans* 2-40 [118].

Two additional GH18 chitinases, MtCh509 [50] and rChi1602 [79] were characterized from *Microbulbifer thermotolerans* DAU221 and *Microbulbifer* sp. BN3, respectively. MtCh509 was expressed in *E. coli*. Some organic solvents (benzene, DMSO, hexane, isoamyl alcohol, isopropyl alcohol, and toluene) increased the reactivity of MtCh509 relative to the aqueous system, representing the first solvent‑tolerant chitinase from *Microbulbifer* species and its potential applications in industrial processes [50]. rChi1602 exhibited maximal activity at 60 °C and over a broad pH range between 5.0 and 9.0 and was highly expressed in *Pichia pastoris* [79].

#### Carrageenase

**Figure 5:**
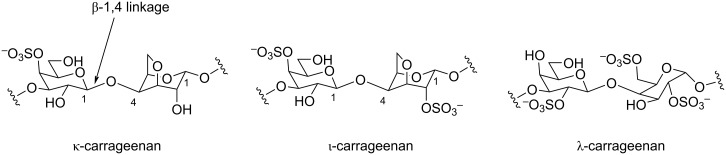
Chemical structures of sulfated polysaccharides κ-, ι-, and λ-carrageenans.

Carrageenans are sulfated saccharide polymers with a wide range of commercial applications. The differential sulfation patterns allow for carrageenans to be differentiated into three basic forms – κ-, ι-, and λ-carrageenans (Figure 5) [28]. Carrageenans find utility in drug and food industries because of their antioxidant and antiviral activities [119–121].

In the sequenced genome of a marine-derived *Microbulbifer thermotolerans* JAMB-A94^T^ was detected an ι-carrageenase encoded by gene *cgiA* [28]. The recombinant CgiA enzyme was produced in *B. subtilis* and its biochemical characterization revealed it exhibited maximal activity at 50 °C. It can break ι-carrageenan into tetrasaccharide at high proportion. A conserved Glu351, rather than an Asp, was identified as the catalytic residue by site-directed mutagenesis experiments.

Another ι-carrageenase, Car1293, was identified, expressed in *E. coli,* and characterized from macroalgae-associated *Microbulbifer* sp. YNDZ01 [89].

#### β-Glucosidases

The β-glucosidases are enzymes that can hydrolyze the β-ᴅ-glycosidic bonds to release glucose at the non-reducing end of oligosaccharides. Based on their substrate specificity, β-glucosidases can be divided into aryl-β-glucosidases that act on aryl glucosides, cellobiases that hydrolyze the disaccharide cellobiose, and other glucosidases [122]. They can also be classified into GH-1 and GH-3 families based on sequence similarities [123].

The first β-glucosidase characterized from *Microbulbifer* bacteria is the GH-3 MtBgl85 derived from the *Microbulbifer thermotolerans* isolated from deep sea sediment [51]. It was expressed in *E. coli.* A gene encoding the GH-1 β-glucosidase MaGlu1A was cloned and expressed in *E. coli* from a marine *Microbulbifer* sp. ALW1 [59]. Enzyme structure and site-directed mutagenesis led to the identification of key residues in the enzyme active site that participated in the hydrolytic activity [59].

#### Carbohydrate esterases

Carbohydrate esterases (CEs) catalyze the *O*- or *N*-deacylation of substituted saccharides. The vast diversity among CEs is organized into 16 different families [123]. Members of the CE6 family are typical serine-type esterases. A cold-adapted CE6 enzyme, CEST, was cloned and expressed in *E. coli* from *Microbulbifer thermotolerans* DAU221 with a temperature optimum of 15 °C [48]. The three-dimensional structure of CEST revealed that it belongs to the α/β-class of proteins consisting of a central six-stranded β-sheet flanked by eight α-helices. Site-directed mutagenesis indicated that a Ser-His-Glu catalytic triad was essential for the enzyme activity [48]. Another cold-adapted CE was isolated from a fosmid genomic library of *Microbulbifer thermotolerans* DAU221 with a similar catalytic triad and expressed in *E. coli* [56].

#### Other enzymes

Chondroitinases (ChSases) are enzymes that digest chondroitin sulfate chains to generate disaccharides. They can be classified into hydrolases and lyases, based on their enzymatic mechanism. ChSase B6 is a chondroitinase originally identified in marine *Microbulbifer* sp. ALW1 [60] and was cloned and expressed in *E. coli.* ChSase B6 could digest chondroitin sulfate B into disaccharides with good thermostability and stability against surfactants. Structural analysis and site-directed mutagenesis identified residues important for the catalytic activity of ChSase B6 [60]. In addition, a novel laminarinase, MaLamNA, was characterized and expressed in *Pichia pastoris* from the same bacterium. It functioned exclusively towards the substrate laminarin and represented the first β-1,3-glucanase from the genus of *Microbulbifer* [58].

*Microbulbifer* sp. JAM-3301 is an inulinase-producing bacterium isolated from deep sea sediment. An inulin operon that contained three open reading frames was cloned and sequenced. One inulinase and one β-fructofuranosidase were expressed in *E. coli* [41]. Both enzymes can work together to effectively degrade inulin.

Lipases are enzymes that can hydrolyze various fatty esters and find applications in the detergent, oil, and pharmaceutical industries. The *lip4346* is a novel lipase-encoding gene cloned from the macroalgae-derived *Microbulbifer* sp. YNDZ01 and expressed in *E coli* [88]. The recombinant enzyme Lip4346 showed high stability at high temperatures and in alkaline conditions, and tolerance to organic solvents.

α-Amylases are glycoside hydrolases that catalyze the hydrolysis of internal α-1,4-*O*-glycosidic bonds in starch and maltodextrins. An α-amylase AmyA from *Microbulbifer thermotolerans* DAU221 was heterologously expressed in *E. coli,* purified, and characterized [49]. It represents the first α-amylase reported from the *Microbulbifer* genus [49].

### Natural products from *Microbulbifer* genus

#### 4-Hydroxybenzoate esters

**Figure 6:**
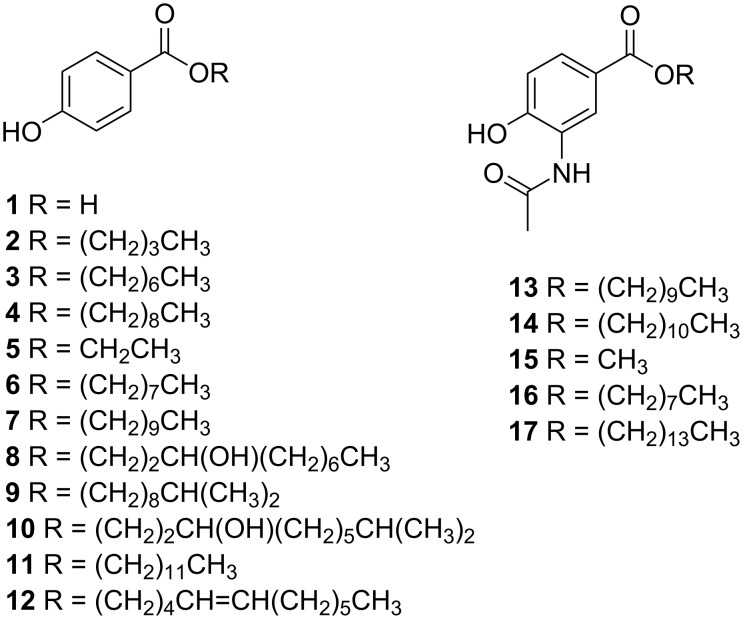
Chemical structures of 4HBA (**1**) and parabens (**2**–**14**) isolated from *Microbulbifer* strains, and synthetic analogus (**15**–**17**).

The first group of secondary metabolites described from the genus *Microbulbifer* were 4-hydroxybenzoate (4HBA, **1**) and three alkyl esters (butyl, heptyl, and nonyl, **2**–**4**, Figure 6). Commonly known as parabens, these molecules were isolated from a tropical ascidian-derived *Microbulbifer* sp. A4B-17, the crude extract of which demonstrated modest antimicrobial activity against several Gram-positive bacteria, molds, and yeasts [6]. The study further characterized another 23 *Microbulbifer* bacteria derived from different marine sources. Although they were able to produce 4HBA in lower amounts, other parabens were not detected.

Parabens are used as preservatives in pharmaceutical, food, and cosmetic industries due to their low toxicity, stability, and activity against a broad spectrum of microorganisms [124]. Industrially, parabens are prepared starting from 4HBA, which in turn, is a petrochemical. *Microbulbifer* sp. A4B-17 constitutes the first microorganism to accumulate 4HBA and parabens, most probably as a metabolite derived from the shikimate pathway [125].

A variety of parabens were isolated and characterized from a marine *Microbulbifer* sp. L4-n2 (Figure 6) [13]. In additon to four known parabens (**2**, **5**, **6**, and **11**), five new ones (**7**–**10**, and **12**) were identified. Their structures were elucidated by high-resolution mass spectrometry (HRMS) and 1D and 2D nuclear magnetic resonance (NMR). The configuration in **8** and **10** remained undetermined. Antimicrobial activities of compounds **2** and **5**–**12** against *Staphlyococcus aureus* indicated that **7** and **12** were bacteriostatic, while **9** and **10** were bactericidal. Compound **9** was also active towards another two commensal Gram-positive bacteria *Bacillus* sp. and *Planococcus* sp. isolated from the host sponge. Compound **9** can be detected in situ in the sponge collected at different times, suggesting it may serve an ecological role in the balance between bacterial community and the sponge host [13].

**Figure 7:**
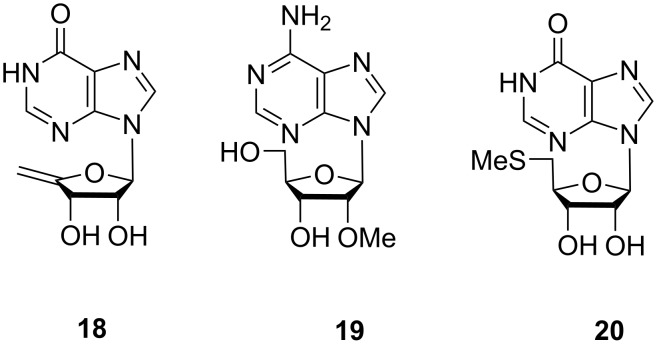
Chemical structures of nucleosides **18**–**20** isolated from *Microbulbifer* strains.

Jayanetti et al. investigated 16 *Microbulbifer* strains isolated from marine tunicate *Ecteinascidia turbinate*. Among these, one strain *Microbulbifer* sp. WMMC-695 showed antimicrobial activity against *E*. *coli* [14]. Bioassay-guided fractionation led to the isolation of two new acetamidobenzoate esters: bulbiferate A (**13**) and bulbiferate B (**14**, Figure 6). Their structures were established on the basis of comprehensive spectroscopic and spectrometric data. Their structures differ from common parabens by the presence of an acetamide group *ortho* to the phenolic hydroxy group [14]. They exhibited weak activity against *E*. *coli* and methicillin-sensitive *Staphylococcus aureus* (MSSA). The three analogues **15**–**17** (Figure 6) with different ester chain lengths were synthesized and tested for antimicrobial activity. However, no clear trend was found between the ester length and bioactivity. In addition to the paraben derivatives, three known nucleosides, 4´,5´-didehydro-5´-deoxyinosine (**18**), 2´-*O*-methyladenosine (**19**), and 5´-methylthioinosine (**20**) were also identified from this bacterium by comparison of their ^1^H NMR and MS data with those reported in literature (Figure 7).

#### Alkaloids

**Figure 8:**
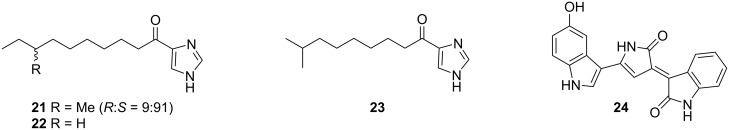
Chemical structures of alkaloids **21**–**24** isolated from *Microbulbifer* strains.

Three new alkanoyl imidazoles **21**–**23** (Figure 8) were isolated from a culture extract of a *Microbulbifer* bacterium isolated from a scleractinian coral [15]. Their structures were determined using a combination of NMR spectroscopy and chemical derivatization experiments, adding new members to this class of imidazole-containing natural products such as the nocarimidazoles A and B reported from a marine-derived actinomycete *Nocardiopsis* sp. before [126]. Compound **21** was determined to be a pair of enantiomers with a ratio of 9% *R* and 91% *S* using the Ohrui–Akasaka method [127]. Structurally, compound **21** represents the second example with a (*R*)-*anteiso* enantiomer in addition to nocapyrone L [128]. An antimicrobial activity assay showed that compounds **21**–**23** could inhibit the growth of Gram-positive bacteria *Kocuria rhizophila* and *S*. *aureus*, Gram-negative bacterium *Tenacibaculum maritimum*, and a panel of fungi. These molecules also demonstrated moderate cytotoxicity against P388 murine leukemia cells.

The bisindole alkaloid violacein (**24**, Figure 8) was isolated and characterized from a sponge-derived *Microbulbifer* strain [73]. This deep purple pigment has been isolated from several other bacterial genera [129], including but not limited to the epiphytic commensal marine bacteria of the genus *Pseudoalteromonas* where the biosynthetic pathway was eventually established [130–131]. The fact that *Microbulbifer* bacteria produce natural products that have been detected to be formed by other marine bacterial genera supports the hypothesis that biosynthetic gene clusters encoding production of natural products with wide ranging implications in bacterial physiology or chemical interactions in the marine environment may be broadly shared [132]. Further examples of shared natural products are delineated below.

#### Aliphatic and aromatic hydrocarbons

**Figure 9:**
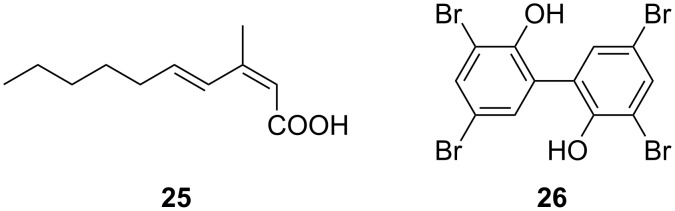
Chemical structures of (2*Z*,4*E*)-3-methyl-2,4-decadienoic acid (**25**) and 4-BP (**26**) natural products isolated from *Microbulbifer* strains.

(2*Z*,4*E*)-3-Methyl-2,4-decadienoic acid (**25**, Figure 9), an unsaturated fatty acid with unusual methylation pattern was isolated and identified from a coral-associated *Microbulbifer* sp. [16]. The molecule was previously known as a synthetic compound; this is the first report of it being detected as a secondary metabolite from a microorganism. Compound **25** is weakly active against *Saccharomyces cerevisiae*. The position of the branching methyl group in **25** is rare. Feeding [1-^13^C]acetate to the culture resulted the enrichment of C1, C3, C5, C7, and C9 carbons, which verified the origin of the carbon skeleton as being fatty acid-derived. Feeding experiments with ʟ-[*methyl*-^13^C]methionine yielded only enrichment of C11 methyl, indicating that this branching methyl could be derived from via *S*-adenosylmethionine (SAM).

Phytoplankton and bacteria have had a long-term coexistence and complex interactions ranging from mutualism, antagonism, and competition to parasitism in the ocean [133]. Algicidal bacteria often emerge in the late stage of an algal bloom and are thought to be involved in the cessation of the bloom [134]. However, molecular algicidal strategies and their global prevalence remain understudied. Recently, Zhang, et al. reported a marine *Microbulbifer* sp. RZ01 which demonstrated broad algicidal activity against 11 cultivated algae [17]. Further investigation indicated that the bioactive substance was mainly located in the cell-free supernatant of *Microbulbifer* sp. RZ01. Fractionation and spectroscopic characterization led to the isolation and identification of the extracellularly secreted molecule, 3,3´,5,5´-tetrabromo-2,2-biphenyldiol (4-BP, **26**, Figure 9).

An in situ algal bloom was simulated to test the algicidal activity of compound **26**. These experiments demonstrated that even low concentrations of **26** can cause changes in overall phytoplankton community. Mechanistic investigations suggested that compound **26** could disrupt electron transport in the photosynthetic light reactions by interfering with the synthesis of plastoquinone-9 [17]. Global genome sequence analyses confirmed the ubiquitous presence of biosynthetic gene clusters (BGCs) encoding the production of **26** in diverse marine bacterial species, suggesting it to be a potent bacterial tool to mediate bacterial–algal antagonistic relationships. Indeed, prior to isolation from a *Microbulbifer* strain, molecule **26** had been isolated from marine *Pseudoalteromonas* and *Marinomonas* genera, and the accumulation and biotransformation of this bacterial natural product in marine mammal metabolomes were postulated [135–137]. In the same vein, before *Microbulbifer*, BGCs encoding production of **26** were also first identified and experimentally characterized from *Pseudoalteromonas* and *Marinomonas* bacteria; genes encoding biosynthetic enzymes that assembled **26** were clustered together with genes encoding the production of polybrominated pyrroles [138–140]. The *Microbulbifer* BGCs lack genes for brominated pyrrole biosynthesis and polybrominated pyrroles have not been reported to be produced by *Microbulbifer* bacteria. Future studies looking at the evolutionary relationship between polybrominated phenol biosynthetic enzymes in diverse marine microbial genera will undoubtedly complement the wide distribution of these natural products in the marine metabolome. All marine bacteria discussed in this section are members of commensal or symbiotic microbiomes of marine invertebrates and plants, perhaps hinting at these molecules being widely used alphabets in inter-organismal chemical cross talk.

#### Non-ribosomally synthesized peptidic natural products

**Figure 10:**
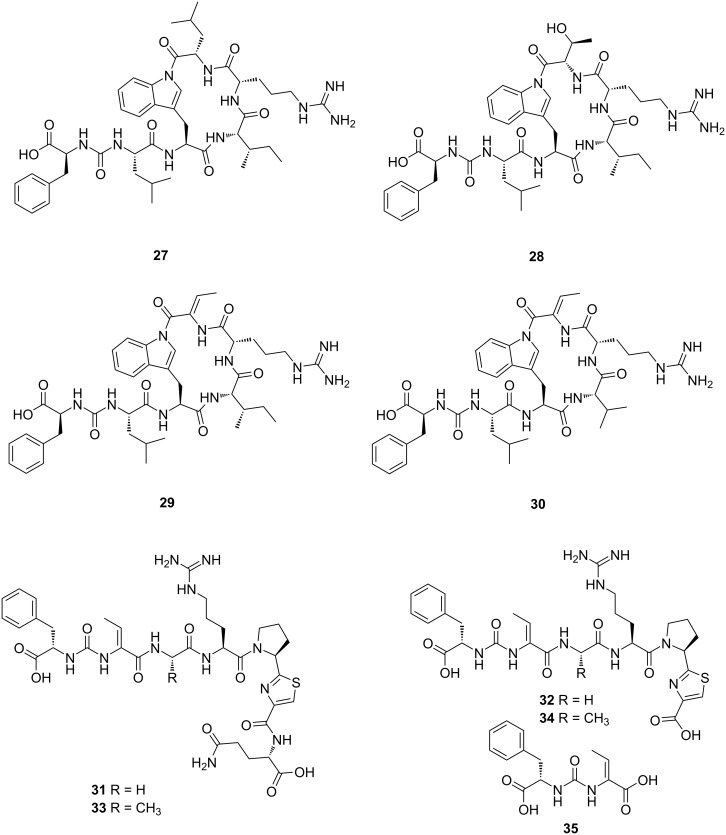
Chemical structures of bulbiferamides **27**–**30** and pseudobulbiferamides **31**–**35**.

Assembly line non-ribosomal peptide synthetases (NRPSs) are routinely detected to be encoded in *Microbulbifer* genomes [141]. An NRPS-derived macrolactam ureidopeptide, bulbiferamide (**27**, Figure 10), was isolated and characterized from a coral-derived *Microbulbifer* sp. C10-1 by Igarashi and co-workers [7]. The ureido linkage did not allow for the peptide main chain in **27** to possess a terminal primary amine and none of the side chains possessed a nucleophilic primary amine. Curiously, the macrocyclizing amide bond was found to exist between a Trp side chain indole nitrogen and the C-terminal carboxylate. To the best of our knowledge, this was the first example of the involvement of a Trp indole in macrolactam formation in bacterial natural products, the only other example being the fungal psychrophilins [142–144]. It should be noted that the Trp indole nitrogen does participate in amide bond formation in other fungal natural products as well, but not in the context of macrocycle assembly [145]. Compound **27** inhibited the growth of *T. cruzi* epimastigotes with an IC_50_ value of 4.1 μM, which is more potent than the approved drug benznidazole (IC_50_ = 20 μM) [7].

Concomitant with Igarashi, we reported the isolation of **27** from a sponge-derived *Microbulbifer* sp. MLAF003 [4]. Three analogues (**28**–**30**, Figure 10) were detected and characterized by NMR and MS from another sponge-derived *Microbulbifer* sp. VAAF005. Additional congeners were detected in the extracts of yet another sponge-derived *Microbulbifer* sp. 22VTAC004. These data demonstrate that the potential for bulbiferamide production is widely spread among *Microbulbifer* bacteria and is not limited to *Microbulbifer* strains isolated from either coral or sponge microbiomes. Imaging mass spectrometry demonstrated that bulbiferamides are excreted by *Microbulbifer* bacterial colonies into the extracellular media.

Based on the characteristic mass spectrometric fragmentations of the Phe-ureido moiety in **27**–**30**, we detected additional ureidopeptidic natural products in the extracts of the sponge-derived strain *Microbulbifer* sp. MKSA007. Subsequent isolation and structure elucidation efforts yielded a new group of ureidopeptides **31**–**35** (Figure 10) which we named pseudobulbiferamides [3]. Congruent with the theme of *Microbulbifer* bacteria sharing natural products with other marine bacteria, compounds **31**–**34** are similar to pseudovibriamides, ureidopeptide natural products that are produced by numerous strains of *Pseudovibrio* bacteria [146]. Moreover, BGCs encoding production of pseudobulbiferamides in *Microbulbifer* and pseudovibriamides in *Pseudovibrio* are quite similar and are located on plasmids, rather than chromosomal DNA. With the observation that *Pseudovibrio* and *Microbulbifer* co-inhabit commensal microbiomes of marine animals, it is tantalizing to speculate that this plasmid borne BGC has been shared. Unlike the bulbiferamides mentioned above, the pseudobulbiferamides were not exclusively excreted out of the bacterial colonies.

### Biosynthesis

**Figure 11:**
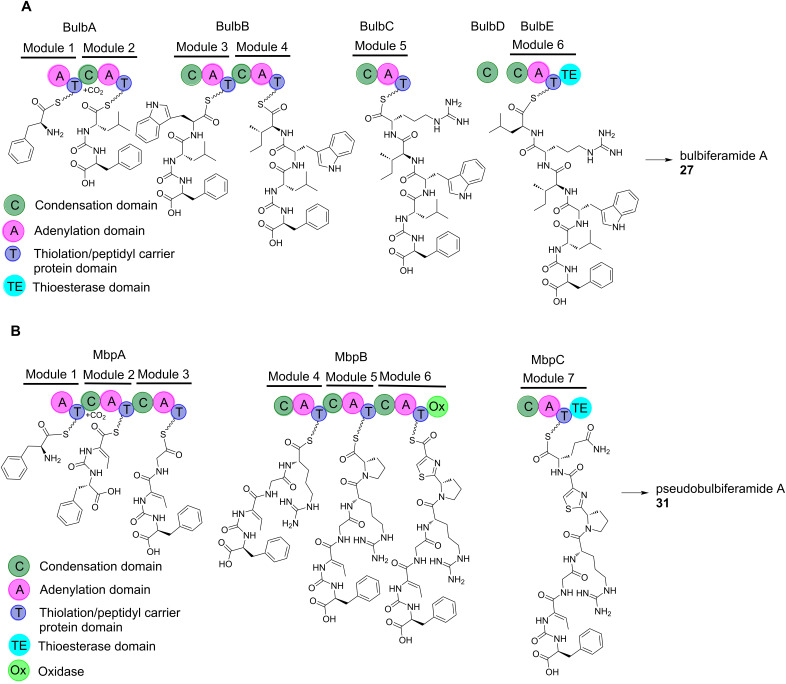
Proposed NRPS assembly lines for the biosynthesis of (**A**) bulbiferamide A (**27**) and (**B**) pseudobulbiferamide A (**31**).

The first genome sequence of a *Microbulbifer* bacterium was reported by Howard et al. in 2003 [117]. Now, more than 70 *Microbulbifer* genomes are publicly available which are typically 5 Mbp in size. Computational mining these genomes with antiSMASH reveals that these bacteria, though not endowed with the biosynthetic prowess of actinomycetes, do still possess BGCs encoding NRPS-derived peptides, polyketides, RiPPs, and siderophores [4,147]. A typical *Microbulbifer* genome contains less than ten BGCs, as identified by antiSMASH, which implies that a large fraction of their genome is not devoted to natural product biosynthesis. The bulbiferamides and the pseudobulbiferamides represent the first and to date the only examples of peptidic natural products isolated from *Microbulbifer* (the corresponding BGCs and NRPS assembly lines are identified as denoted in Figure 11) [3–4]. Thus, it is immediately apparent that the potential for novel compound discovery and for the heterologous production of cryptic natural products from this genus is high. Correlating *Microbulbifer* genomes and metabolomes will drive advances in these directions.

**Figure 12:**
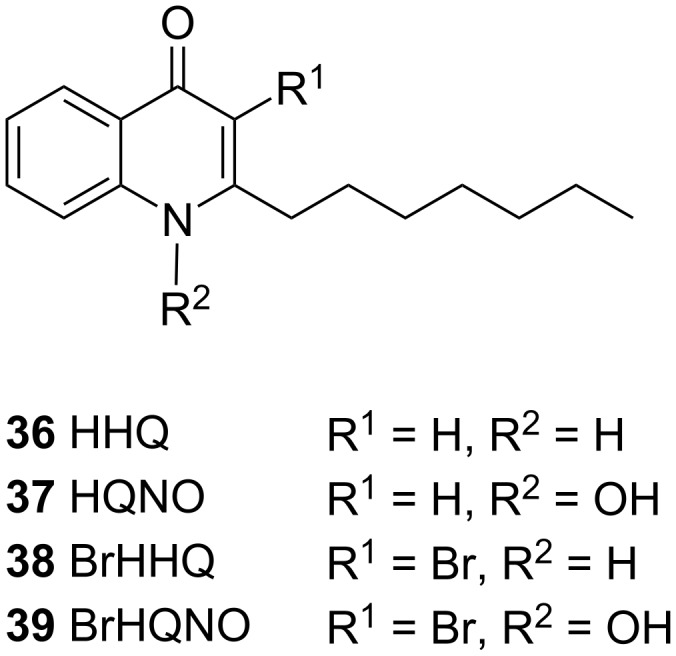
Chemical structures of 2-heptyl-1*H*-quinolin-4-one (**36**, HHQ), 2-heptyl-1-hydroxyquinolin-4-one (**37**, HQHO), and their brominated products BrHHQ **38** and BrHQHO **39**.

The genome sequence of *Microbulbifer* sp. strain HZ11 revealed the presence of an alkylquinolone (AQ)-producing BGC; however, no AQs were detected to be produced by this strain. This BGC was unique to strain HZ11 among the available *Microbulbifer* genomes [148]. Feeding AQ substrates 2-heptyl-1*H*-quinolin-4-one (**36**, HHQ, Figure 12), 2-heptyl-1-hydroxyquinolin-4-one (**37**, HQHO) to *Microbulbifer* sp. strain HZ11 yielded the brominated AQ products BrHHQ **38** and BrHQHO **39**. A gene encoding a vanadium-depenent haloperoxidase (VHPO) was detected in the genome and recombinantly produced VHPO was shown to brominate AQs. Besides, the brominated AQs demonstrated increased antibiotic activity against *Staphylococcus aureus* and other marine bacteria [148]. In addition to the antimicrobial activity demonstrated herein, AQs are fundamentally signaling molecules and enzymatic modification of AQs by *Microbulbifer* could represent a mechanism of ecological cross-talk among marine microbiomes. Enzymatic halogenation of other signaling molecules, such as that of acyl homoserine lactones, has been postulated to modulate bacterial interactomes [149–150].

## Conclusion

Geographically, halophilic *Microbulbifer* bacteria are widely distributed with marine sponges (40.4%) and sediments (14.7%) being the most prolific sources for isolation (Figure 1, Table 1). Twelve groups of degradation enzymes have been expressed and characterized. A total of 32 natural products, including NRPSs, PKS, parabens, and alkaloids, have been isolated and identified from *Microbulbifer* bacteria. Based on the growing genomic data, it is unquestionable that the *Microbulbifer* genus is an untapped resource for natural products. The number of molecules described thus so far is not comparable to the expected metabolites envisioned from bioinformatics analyses of the *Microbulbifer* genomes. Contemporary and in-development tools and technologies for data mining, synthetic biology, and strain manipulation will have a transformative effect on future natural product discovery from the *Microbulbifer* genus.

## Data Availability

Data sharing is not applicable as no new data was generated or analyzed in this study.
